# Mimicking sMMOH chemistry: trapping the Sc^3+^-bound nonheme Fe^III^–O–O–Fe^III^ adduct prior to its conversion into an Fe^IV^_2_(μ-O)_2_ core

**DOI:** 10.1039/d5sc05667e

**Published:** 2025-10-03

**Authors:** Patrick M. Crossland, Bittu Chandra, Saikat Banerjee, Chase S. Abelson, Yisong Guo, Marcel Swart, Lawrence Que

**Affiliations:** a Department of Chemistry, University of Minnesota Minneapolis MN 55455 USA larryque@umn.edu; b Department of Chemistry, Carnegie Mellon University Pittsburgh PA 15213 USA ysguo@andrew.cmu.edu; c IQCC and Department of Chemistry, University of Girona 17003 Girona Spain marcel.swart@udg.edu; d ICREA Pg. Lluís Companys 23 08010 Barcelona Spain

## Abstract

Di-iron systems that activate O_2_ to form high-valent, oxo-bridged Fe^IV^_2_ or Fe^III^Fe^IV^ products are of great interest to bio-inorganic chemists due to their relevance to the chemistry of soluble methane mono-oxygenase (sMMOH), which incorporates both atoms of O_2_ gas into a diiron(iv) complex with an Fe_2_O_2_ diamond core. In this study, the [Fe^III^_2_(Me_3_NTB)_2_(μ-O)(μ-O _2_)]^2+^ adduct (Me_3_NTB = tris((1-methyl-1*H*-benzo[*d*]imidazole-2-yl)methyl)amine) reacts with two Sc^3+^ to break the O–O bond that in turn forms the target Fe^IV^(μ-O)_2_Fe^IV^ product. This study provides the first evidence that a Lewis acid can interact directly with a diferric-peroxo complex to initiate O–O bond cleavage, as evidenced *via* vibrational and X-ray absorption spectroscopy.

## Abstract 2

The data in this paper represent the first documented evidence for Lewis-acid adduct formation to a 1,2-peroxodiferric species derived from the reaction of a diiron(ii) complex with O_2_ en route to its conversion into an Fe^IV^_2_(μ-O)_2_ diamond core.

## Graphical abstract

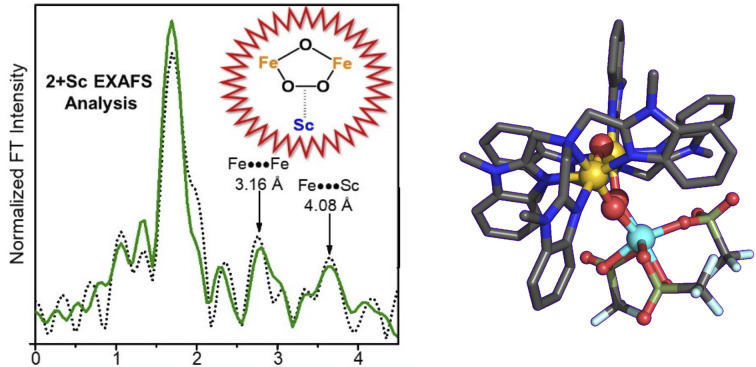

## Introduction

**Scheme 1:**
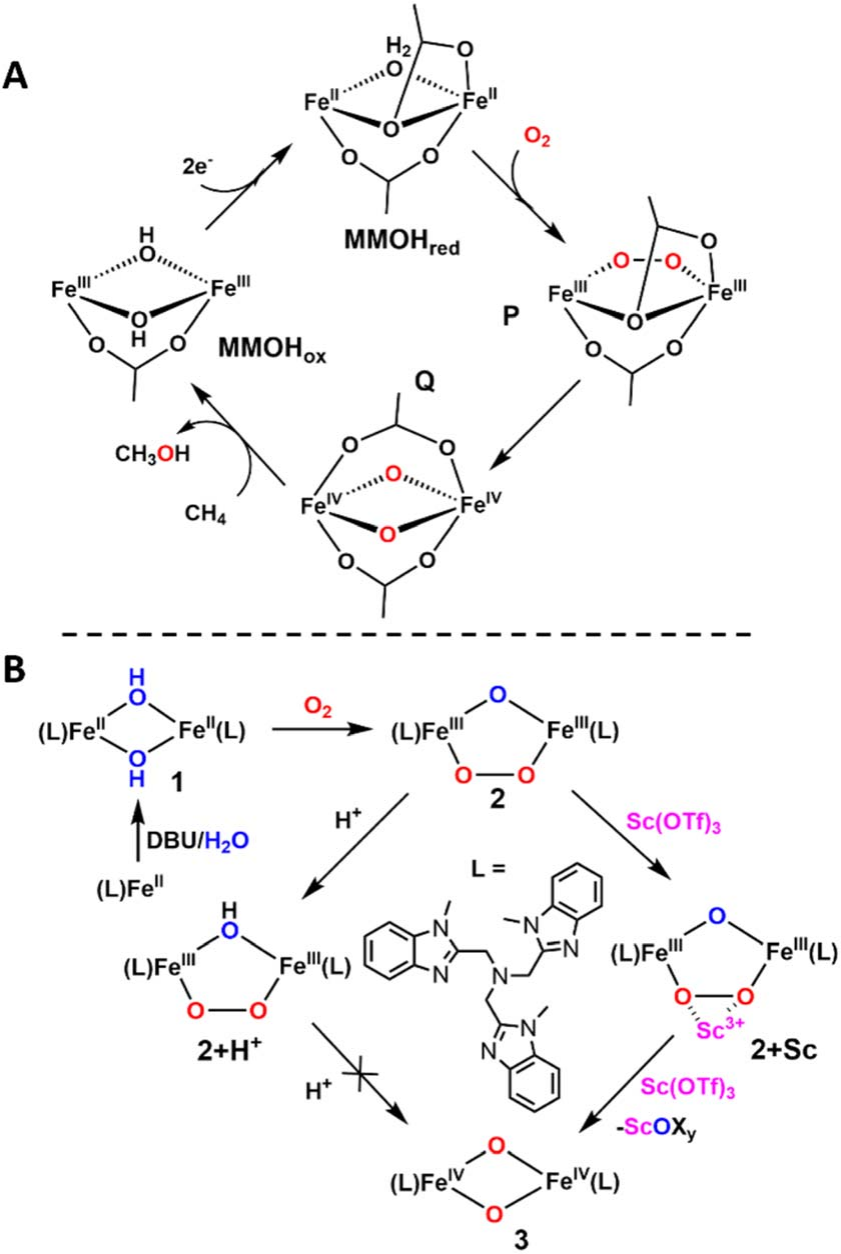
(A) Proposed catalytic cycle of sMMO and (B) generation of intermediates 2, 2+H^+^, 2+Sc, and 3 in the biomimetic system.

In Nature, nonheme diiron enzymes have been found to activate dioxygen to perform a variety of reactions like the hydroxylation of methane and the eukaryotic initiation factor 5a, the oxidative desaturation of fatty acids, as well as the conversion of ribonucleotides to deoxyribonucleotides.^1^ Of particular interest is the soluble methane monooxygenase hydroxylase enzyme (sMMOH), which hydroxylates the very strong C–H bond of methane (C–H BDFE = 105 kcal mol^−1^) to form methanol.^2^ As shown in Scheme 1A, the catalytic cycle of sMMOH is initiated at its diferrous site, to which dioxygen binds to form intermediate P, which is best described as a diferric-peroxo species.^3^P then converts into the diiron(iv) intermediate Q in a proton-dependent step^3,4^ that results in O–O bond cleavage and the incorporation of both atoms of O_2_ into this key intermediate.^5^

Modeling this reaction has been a goal for synthetic inorganic chemists, and various diiron compounds have been used to mimic the proposed intermediates along the dioxygen activation pathway of sMMOH.^6–17,26,27^ Several diferric-peroxo intermediates have been identified that convert into higher-valent species by treatment with acid.^15–17^

More recently, we have reported a biomimetic system based on a mononuclear Fe^II^(Me_3_NTB) precursor (Me_3_NTB = tris((1-methyl-1*H*-benzo[*d*]imidazole-2-yl)-methyl)amine) that forms a diiron(ii) derivative [(Me_3_NTB)Fe^II^(μ-OH)_2_Fe^II^(Me_3_NTB)]^2+^ (1) upon treatment with a suitable base, and is converted upon exposure to dioxygen into a (μ-oxo)(μ-1,2-peroxo)diiron(iii) species 2 ([(Me_3_NTB)Fe^III^(μ-O)(μ-1,2-O_2_)Fe^III^(Me_3_NTB)]^2+^). This diferric species in turn transforms into the diferryl diamond core complex 3 ([(Me_3_NTB)Fe^IV^(μ-O)_2_Fe^IV^(Me_3_NTB)]^4+^) upon treatment with Sc(OTf)_3_.^16^ In this study, we report the characterization of a previously unidentified short-lived intermediate that forms immediately upon addition of Sc^3+^ to a solution of 2 in the course of its conversion into 3 (Scheme 1B). The fleeting 2+Sc complex represents the first biomimetic example of a diferric-peroxo complex to be trapped with a bound Lewis acid prior to its conversion into a diiron(iv) complex having an Fe^IV^_2_(μ-O)_2_ diamond core.

## Results and discussion

### Synthesis and UV-Vis characterization of 2+Sc complex

**Fig. 1:**
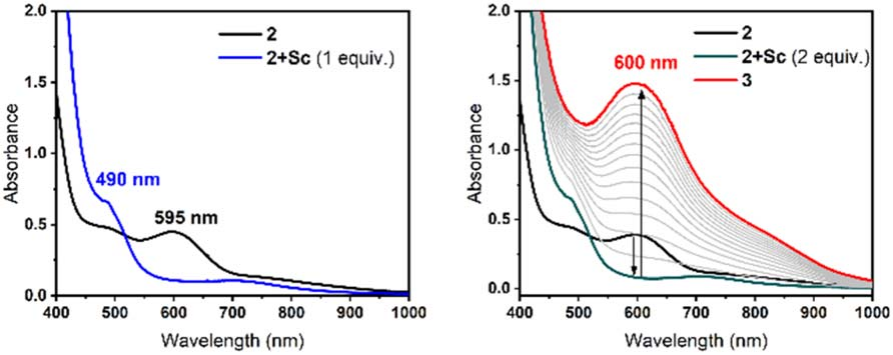
UV-Vis spectral changes at −40 °C upon treating 0.50 mM 2 in MeCN with 1 equiv. Sc^3+^ to generate 2+Sc (left, blue line) and with 2 equiv. of Sc^3+^ (right, cyan line) *en route* to its conversion to 3 (spectral changes after adding 2 equiv. Sc^3+^, right, spectra taken every 5 minutes).

The generation and characterization of (μ-oxo)(μ-1,2-peroxo)diiron(iii) complex (2) has been previously reported by our group (Scheme 1B).^16^ Treatment of a solution of 2 in MeCN at 233 K with 1 equiv. Sc(OTf)_3_ (or, Sc^3+^) results in an immediate change in its UV-Vis features (Fig. 1 and S5), forming a species designated as 2+Sc with a peak at *ca.* 350 nm, and a less intense visible band at *ca.* 490 nm. These peaks are blue-shifted relative to 2, which showed bands at *ca.* 400 nm and 595 nm.^16^ An identical UV-Vis spectrum (Fig. S1) is generated directly upon the addition of 2 equiv. Sc^3+^ to 2. However, over the course of an hour, this addition of a second equivalent of Sc^3+^ results in a red-shift to *ca.* 400 nm and *ca.* 600 nm for the less intense band (corresponding to 3). Clearly, one equiv. Sc^3+^ is sufficient to generate the 2+Sc adduct, but adding the second equivalent of Sc^3+^ to this solution is required to generate 3. These observations suggest that the first Sc^3+^ ion binds to the O–O bond, and only after the addition of the second Sc^3+^ ion does O–O bond cleavage occurs.

Similar blue shifts (as observed for the change from 2 to 2+Sc) have previously been observed in the UV-Vis features of mononuclear Fe^III^–O_2_^2−^ complexes upon treatment with Lewis acids,^18–20^ as well as in di-copper-peroxo systems that bind exogenous Lewis acids.^21–23^ This is in contrast to the red-shift typically found upon addition of protic acids to diferric-peroxo intermediates that result from protonation of the bridging oxo moiety.^15,16^ The opposite effects of protic and Lewis acids on the peroxo-based UV-Vis feature strongly suggest that the blue shift of the peroxo band of 2 upon Sc^3+^ addition comes from the interaction of the Sc^3+^ ion with the peroxo moiety (and not with the oxo bridge), resulting in a decrease in the basicity of the peroxo ligand that promotes the cleavage of the O–O bond.

Crucially, 2+Sc persists for a few seconds at 233 K before undergoing a rearrangement over an induction period of *ca.* 10–15 minutes (Fig. S5), eventually converting into 3 (Fig. 1, right side). This time-lapse allows 2+Sc to be trapped by rapid freeze-quenching for characterization by a variety of spectroscopic methods. While one equivalent of Sc^3+^ generates 2+Sc (as observed by UV-Vis absorption, Fig. 1, left), the addition of the second equivalent allows us to follow the conversion of 2 to 3 by spectroscopic methods.

### Resonance Raman spectroscopy

**Fig. 2:**
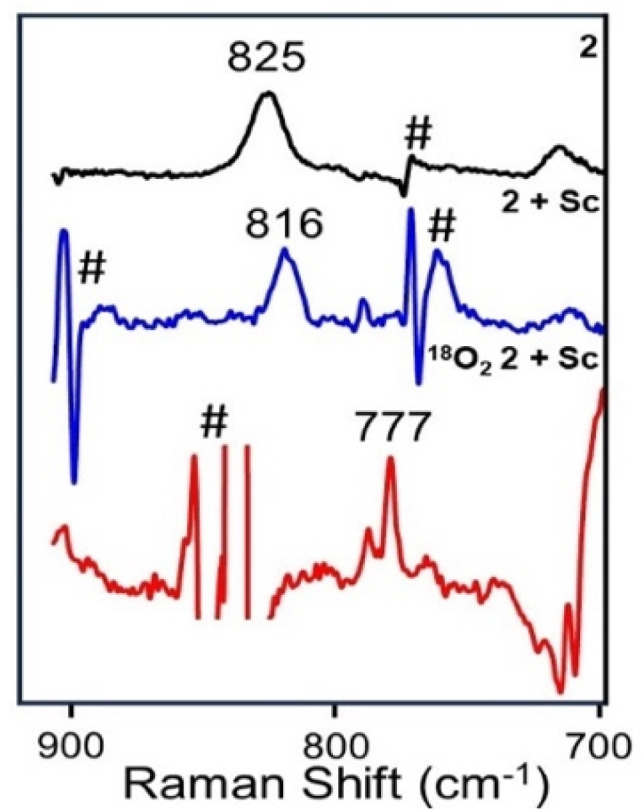
Resonance Raman spectra at 77 K with 135° backscattering of 2 (561 nm excitation) and 2+Sc (457 nm excitation). Black: 0.5 mM ^16^O_2_-2 in MeCN; Blue: 0.5 mM ^16^O_2_-2+Sc in MeCN; Red: 0.5 mM ^18^O_2_-2+Sc in CD_3_CN. # indicates artifacts from solvent subtraction.

Resonance Raman spectroscopy has proven to be a useful probe for the features found in the visible spectra of 2 and 2+Sc (Fig. 2). The O–O vibration for 2 is observed at 825 cm^−1^ and red-shifts by 9 cm^−1^ to 816 cm^−1^ upon addition of Sc^3+^, reflecting the interaction of Sc^3+^ with the 1,2-peroxo bridge. To observe the corresponding O–O vibration of 2+Sc requires irradiation with a 457 nm laser, due to the blue shift in the peroxo-to-iron charge transfer band upon Sc binding. No resonance-enhanced peaks are observed with the use of a 516 nm laser, as used to characterize 2. The red-shift in the O–O vibration for 2+Sc is about half as large as that found for Sc^3+^ binding to [Fe^III^(TMC)(η^2^-O_2_)]^+^ from 826 to 807 cm^−1^,^19^ reflecting the higher basicity of the side-on bound peroxo moiety in the latter complex.

Interestingly, four (Fe–O)-based vibrations observed in 2 (at 715, 527, 518, and 454 cm^−1^)^16^ disappear upon Sc^3+^ addition (Fig. S6), namely the *ν*_sym_(Fe–O_2_–Fe), *ν*_asym_(Fe–O_2_–Fe), *ν*_sym_(Fe–O–Fe), and *ν*_asym_(Fe–O–Fe) features, leaving the O–O stretch at 825 cm^−1^ (red-shifted to 816 cm^−1^) to be the only vibration observed in its resonance Raman spectrum. This dramatic change shows Sc^3+^ binding to 2 to induce a structural alteration significantly distinct from protonation, leading to a distortion of the relatively planar Fe^III^(μ-O)(μ-1,2-O_2_)Fe^III^ moiety that disrupts the well-established vibrational patterns associated with such cores.^6,7,15,16^

The interaction of Sc^3+^ with 2 differs substantially from that of adding a proton to the oxo bridge of 2, for which Raman vibrations at 820 [*ν*(O–O)], 527 [*ν*_asym_(Fe–O_2_–Fe)], and 449 [*ν*_sym_(Fe–O_2_–Fe)] cm^−1^ are observed, the latter two being associated with deformations of the Fe–O–O–Fe unit in 2+H^+^.^16^

**Fig. 3:**
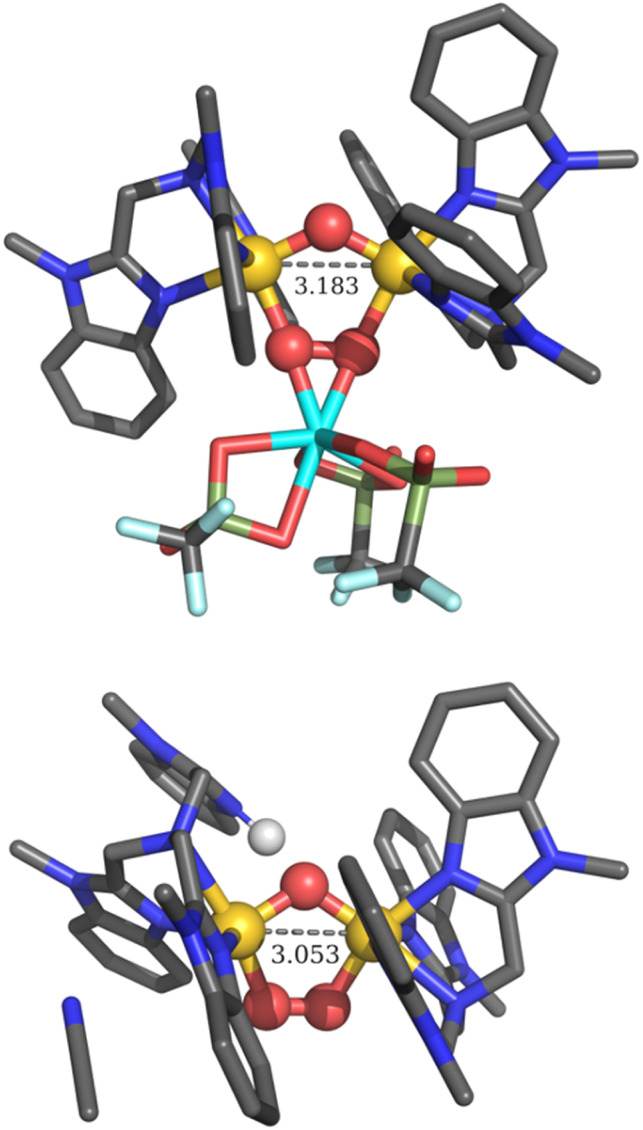
Top: DFT optimized structure of 2+Sc. Bottom: DFT computed structure (r2scan-3c, COSMO) for 2+H^+^, which exhibits the short Fe⋯Fe distance as previously inferred from EXAFS analysis.^16^

Note that our DFT calculations predict that the proton does not bind to the oxo bridge, but instead protonates one of the benzimidazole rings (Fig. 3, bottom). All attempts to optimize structures of 2 with a protonated oxo bridge lead to an Fe–Fe distance of 3.38 Å, or even an opened up [(L)(OH)Fe^III^(μ-1,2-O_2_)Fe^III^(L)(MeCN)]^3+^ complex with an Fe⋯Fe distance of 4.39 Å. Instead, protonation of one of the benzimidazoles affords an Fe–Fe distance of 3.05 Å, leading to computed ν(O–O), *ν*_asym_(Fe–O_2_–Fe), and *ν*_sym_(Fe–O_2_–Fe) vibrations that nearly match those of 2 itself.^12^

The 2+Sc sample obtained using ^18^O_2_ in CD_3_CN shows three peaks at 785, 777, and 760 cm^−1^ in place of the sole feature at 816 cm^−1^ found in the corresponding ^16^O_2_ experiment in CH_3_CN. As the features at 760 cm^−1^ and 785 cm^−1^ can also be observed as shoulders to solvent-derived peaks in the ^16^O spectrum obtained in CH_3_CN shown in Fig. 2, only the vibration at 777 cm^−1^ can be associated with the labeled O–O vibration of 2+Sc. In addition, there is a broad peak at 690 cm^−1^ that could arise from an Fe–O–Fe vibration,^1,24^ but this feature persists even in the spectrum of thermally-decayed 2+Sc and thus must arise from some other byproduct.

### Mössbauer and X-ray absorption spectroscopy

**Fig. 4:**
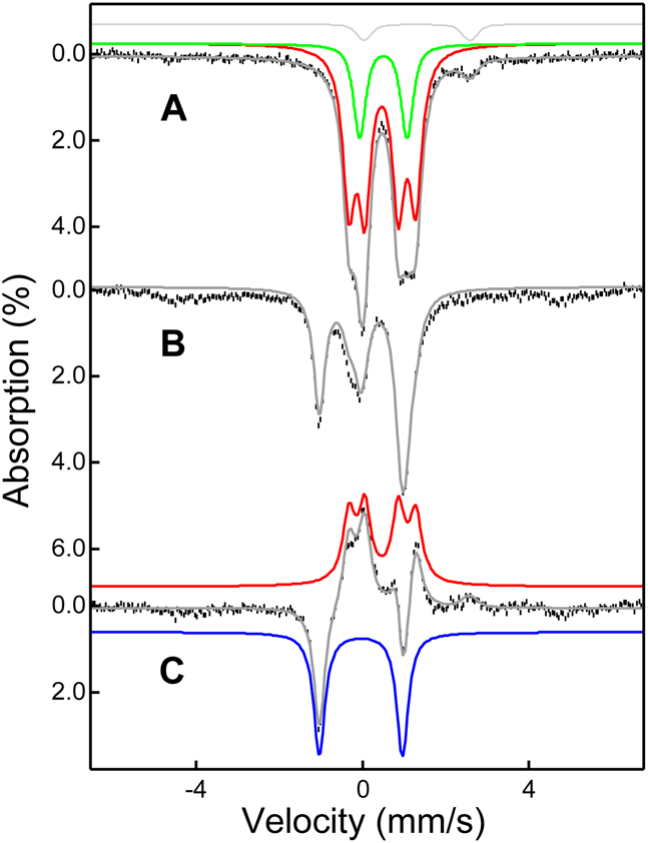
4.2 K zero field Mössbauer spectra (vertical bars) of 2+Sc (panel A) and upon conversion of 2+Sc into 3 (panel B). Panel C shows the B – A difference spectrum highlighting the conversion of 2+Sc to 3. Panel A shows an overall simulation (grey solid line) with four species. The red solid line containing two distinct ferric doublets of equal intensity represents 74% of the iron in the sample and is assigned to the dominant 2+Sc species with distinct Fe^III^ units. The green solid line represents 18% of the iron in the sample corresponding to a symmetric diferric species. The light grey line represents a minor ferrous species (∼5%), likely to be the starting ferrous complex. Panel B shows an overall simulation (grey solid line) with 50% 3, 25% 2+Sc and 18% **2′**. Panel C shows the conversion of 2+Sc (red solid line) into 3 (blue solid line).

Mössbauer spectroscopy shows that the 2+Sc adduct (Fig. 4) differs significantly from previously reported 2.^16^ Complex 2 exhibits a single quadrupole doublet with *δ* = 0.49 mm s^−1^ and Δ*E*_Q_ = 1.06 mm s^−1^, indicating a twofold-symmetric anti-ferromagnetically coupled diferric center. In contrast, 2+Sc exhibits a prominent pair of doublets with comparable intensities that can be associated with an antiferromagnetically coupled pair of high-spin diferric centers, with *δ* = 0.48 mm s^−1^ and Δ*E*_Q_ = 1.60 mm s^−1^ (for 2+Sc A) and *δ* = 0.46 (*A*) mm s^−1^ and Δ*E*_Q_ = 0.81 mm s^−1^ for (2+Sc B), together representing 74% of the total Fe in the sample. There is also a third diferric type quadrupole doublet 2’ representing 18% of the total Fe, with parameters *δ* = 0.50 mm s^−1^ and Δ*E*_Q_ = 1.14 mm s^−1^ that are similar but distinct to those of 2. However, unlike 2+Sc A and 2+Sc B, **2′** does not convert to 3, as shown in Fig. 4C, which leads us to tentatively assign this minor di-ferric type species as a minor byproduct of little relevance to the chemistry of interest, and most likely no longer contains Sc.

Complex 2+Sc shows a K-edge X-ray absorption near edge (XANES) feature at an energy that is 0.6 eV blue-shifted relative to that of 2 (Fig. S4), indicating a lower electron density on the ferric ions. Such a blue-shift may result from the decreased interaction between the O_2_^2−^ ligand and Fe^3+^ ions due to competition with Sc^3+^ binding. Its pre-edge area decreases only slightly upon Sc^3+^ binding, from 18.2 units to 17.5 units (see Fig. S3 in SI), reflecting only a slight change to the symmetry around the iron center, in contrast to the larger effect of adding H^+^ to 2. Although 2+H^+^ has a K-edge energy of 7126.7 eV that is intermediate between those of 2 and 2+Sc, its pre-edge area is significantly smaller due to the weakening of the Fe–oxo bonds upon protonation. In addition, the pre-edge region of 2+H^+^ is best fitted with two features, indicating a significant differentiation between the two (Brønsted/Lewis) acid complexes of 2. Our DFT study reproduces this change in the number of features when going from 2 to 2+H (Fig. S7). Clearly, 2 is affected quite differently by its interactions with H^+^ and Sc^3+^.

**Fig. 5:**
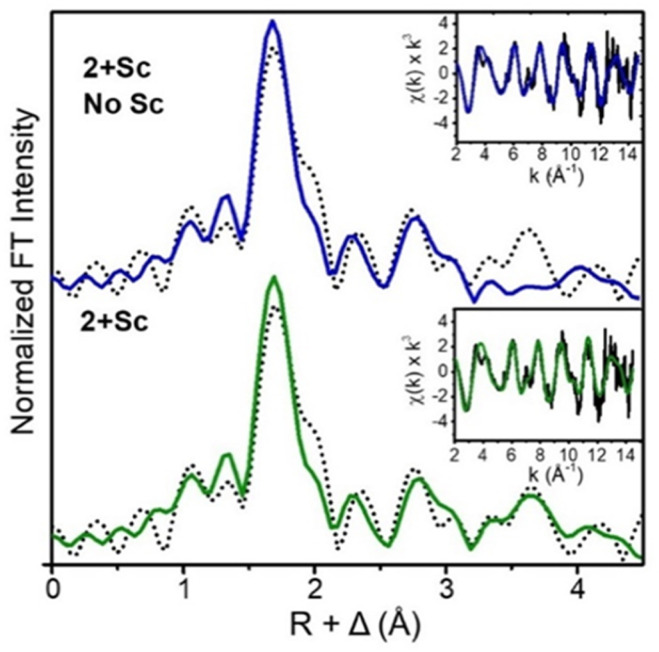
Fourier transformed EXAFS and *k*-space EXAFS (insets) spectra of 2+Sc, with the Sc scatterer at 4.08 Å included in the green colored spectral fits.

As expected, the structure for 2+Sc, as derived from EXAFS analysis (Fig. 5), significantly differs from those of both the parent complex and the protonated complex 2+H^+^. The observed Fe⋯Fe distance lengthens to 3.15 Å (*vs.* 3.07 and 3.09 Å for 2 and 2+H^+^, respectively), and a Sc scatterer is found at 4.08 Å. When the latter is excluded from the fit, there is a prominent unassigned peak in the Fourier-transformed EXAFS spectrum, lending strong support for a Sc^3+^-bound peroxo moiety. While it is possible that a scatterer at this distance could arise from a multiple scattering pathway in the imidazole of the ligand backbone, careful analysis of the EXAFS data precludes such a conclusion. The goodness of fit of the EXAFS data with a carbon shell at the same distance as the Sc shell (with no Sc included) is noticeably worse (Table S2, fit 6 compared to fit 5). Additionally, no such ligand-derived scatterers were observed in the initial report of 2.^16^ DFT calculations support the results of this analysis, predicting an Fe⋯Fe distance of 3.183 Å and Fe⋯Sc distances of 4.10 Å (Fe1⋯Sc) and 4.01 Å (Fe2⋯Sc), in excellent agreement with experimental results. The Sc⋯O distances in 2+Sc are also slightly different, at 2.04 Å (O1⋯Sc) *vs.* 2.08 Å (O2⋯Sc).

### Comparison with literature results

**Fig. 6:**
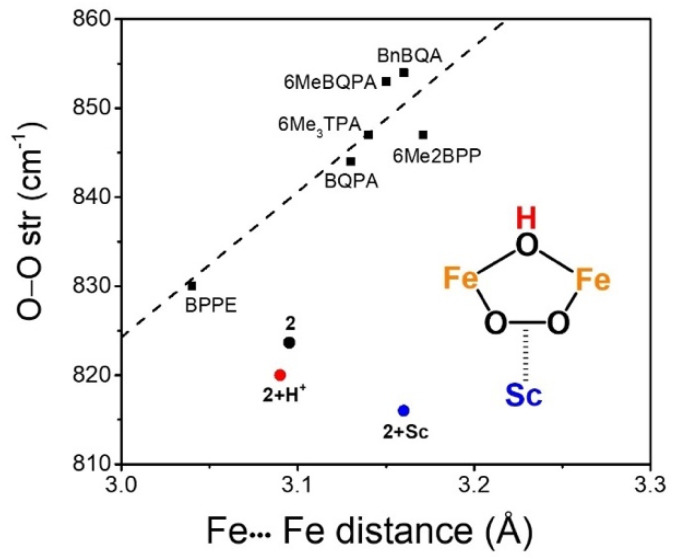
Empirically derived correlation between O–O vibrational frequencies for nonheme (μ-oxo)diferric-peroxo intermediates and their diiron distances as deduced by EXAFS analysis. All points representing the oxo/peroxo complexes fall close to the dashed line. Compared to the other complexes shown, 2 falls furthest away from the dashed line, but even larger deviations are found for 2+H^+^ and 2+Sc, reflecting more significant structural alterations relative to that of 2.

To the best of our knowledge, this study provides the first documented example of a Lewis acid adduct to a diferric–peroxo complex. To put this novel family of complexes into perspective, we compare their properties with those observed for other (μ-1,2-peroxo)diferric complexes in the literature.^6^ In previous work, our laboratory has noted a correlation between the diiron distance and the O–O vibrational frequency (Fig. 6). Within this context, the parent complex 2 has parameters that place it closest to the established trendline among the four related complexes derived from 2. 2+H^+^ falls further away and 2+Sc is even further. Clearly, an even more drastic structural change occurs upon Sc^3+^ binding, which is also reflected by the loss of all Fe–O vibrations (Fig. 2). This distinct behavior likely reflects the loss of the relatively planar [Fe^III^_2_(μ-O)(μ-1,2-O_2_)] framework found in 2 and emphasizes the unique role Sc^3+^ plays in promoting O–O bond cleavage.

The observed activation of the O–O bond by Sc^3+^ in this work stands in sharp contrast to the effect of adding a proton observed for other diferric-peroxo systems. For complexes supported by BnBQA (= *N*-benzyl-1-(quinolinyl)-*N*-(quinolinylmethyl)methanamine)) and 6-Me_2_-BPP (= bis(((6-methylpyridinyl)methyl)-glycine), protonation of their respective oxo bridges results in higher frequency O–O vibrations (854 *vs.* 928 cm^−1^ and 847 *vs.* 908 cm^−1^, respectively).^7,15^ Protonation also does not lead to the formation of diiron(iv) species for the BnBQA and the 6Me_3_TPA (= tris((6-methylpyridinyl)methyl)amine) complexes. Instead, both complexes simply decay upon addition of a protic acid to form Fe^III^Fe^IV^ products. In contrast to what is observed upon protonation in the BnBQA and 6Me_3_TPA systems, 2 converts into 3 upon addition of Sc^3+^, promoting O–O bond scission to generate the Fe^IV^_2_(μ-O)_2_ diamond core of 3.

This outcome parallels the behavior observed for mononuclear [Fe^III^-*η*_2_-O_2_] complexes, which undergo O–O bond scission upon treatment with Lewis acids, leading to the subsequent formation of Fe^IV^ = O products with the help of an electron donor.^18,19,25^ This is not the case in the diferric system 2, where the two required reducing equivalents needed to fully activate O_2_ come from both irons. It was initially proposed, based on resonance Raman labeling experiments, that the Sc^3+^ ion scavenged the bridging oxo to allow for the dinuclear core to isomerize to a form suited for O–O cleavage. However, based on our current results, Sc^3+^ in fact plays a crucial bifunctional role by first priming the O–O bond for cleavage and subsequently scavenging the bridging oxo ligand.

The O–O bond in monoferric–peroxo complexes would appear to be more activated by binding Lewis acids than that of 2+Sc. For example, in the case of [(TMC)Fe^III^(*η*_2_-O_2_)]^+^ (TMC = 1,4,8,11-tetramethyl-1,4,8,11-tetraazacyclotetradecane), the O–O vibration shifts from 826 cm^−1^ to 807 cm^−1^ upon Sc^3+^ binding,^19^ which is almost twice as pronounced as the 10 cm^−1^ red shift observed upon the conversion of 2 to 2+Sc. The Fe⋯Sc^3+^ distance also differs significantly between mono- and diferric systems. EXAFS analysis of the [(TMC)Fe^III^(μ-*η*_2_:*η*_2_-O_2_)Sc]^4+^ adduct reveals an Fe⋯Sc^3+^ distance of 3.84 Å, which concurs with the 3.80 Å distance derived from DFT,^18^ but is significantly shorter than the 4.08 Å distance found for 2+Sc by EXAFS analysis. Although this difference between mono- and diferric systems is not unexpected, it highlights the geometric constraints imparted by the dinuclear system.

## Conclusions

The data presented in this paper represent the first documented evidence for Lewis-acid adduct formation to a 1,2-peroxodiferric species derived from the reaction of a synthetic diiron(ii) complex with O_2_ en route to its conversion into an Fe^IV^_2_(μ-O)_2_ diamond core. This sequence of transformations mimics the steps involved in the conversion of the diiron(ii) active site of soluble methane monooxygenase hydroxylase (sMMOH) upon exposure to O_2_, namely the initial formation of the 1,2-peroxo-diferric derivative P and its subsequent conversion into the diiron(iv) oxidant Q. Our results thus provide strong evidence for the formation of a Sc^3+^ adduct to the peroxo intermediate prior to inducing O–O bond scission to form a diiron(iv) complex with an Fe_2_(μ-O)_2_ diamond core.

## Abbreviations

sMMOH: Soluble methane monooxygenase
BDFE: Bond dissociation free energy

## Author contributions

Patrick M Crossland and Bittu Chandra contributed equally to this work. The manuscript was written through contributions of all authors. All authors have approved the final version of the manuscript.

## Conflicts of interest

There are no conflicts to declare.

## Supplementary Material

SC-016-D5SC05667E-s001

## Data Availability

The computational data have been uploaded to iochem-bd at https://doi.org/10.19061/iochem-bd-4-90, all the other data are available in the supplementary information (SI). Supplementary information: additional XAS data, experimental procedures, and methods. See DOI: https://doi.org/10.1039/d5sc05667e.
