# Supplementary material for: Mimicking sMMOH chemistry: trapping the Sc3+-bound nonheme FeIII–O–O–FeIII adduct prior to its conversion into an FeIV2(μ-O)2 core
Source: Chem Sci. 2025 Oct 3;16(42):19608–13. doi: 10.1039/d5sc05667e (PMC12495302; doi:10.1039/d5sc05667e)
Supplement: SC-016-D5SC05667E-s001 [file SC-016-D5SC05667E-s001.pdf]

Supplementary Information (SI) for Chemical Science.
This journal is © The Royal Society of Chemistry 2025

Supporting information for

Mimicking sMMOH Chemistry: Trapping the Sc^{3+}-bound Nonheme Fe^{III}-O-O-Fe^{III} Adduct Prior to Its Conversion into an $Fe^{IV}_2(\mu\text{-}O)_2$ Core

Patrick M Crossland,[a,d,#] Bittu Chandra,[a,#] Saikat Banerjee,[a] Chase S. Abelson,[a] Yisong Guo[b,*], Marcel Swart,[c,*] Lawrence Que, Jr.[a,*]

[a] Department of Chemistry
University of Minnesota
Minneapolis, MN 55455 (USA)
E-mail: larryque@umn.edu

[b] Department of Chemistry
Carnegie Mellon University
Pittsburgh, PA 15213 (USA)

[c] IQCC and Department of Chemistry
University of Girona
17003 Girona, Spain
ICREA
08010 Barcelona, Spain

[d] Current address: Dept. of Chemistry, University of Chicago, Chicago, IL 60637 (USA)

# These authors contributed equally

Experimental Details

Materials

All reagents and solvents were purchased from commercial sources and used as received unless specified. All moisture- and oxygen-sensitive compounds were prepared using standard Schlenk–line techniques. $[Fe^{II}(Me_3NTB)(CH_3CN)](CF_3SO_3)_2$ (**1**) was synthesized as previously reported.[1] The formation of **2** was accomplished as previously reported.[2]

Methods

UV-vis absorption spectra were recorded on an HP 8453A diode array spectrometer. Low-temperature visible spectra were obtained using a cryostat from UNISOKU Scientific Instruments, Japan. Raman spectra were collected with an Acton AM–506 monochromator equipped with a Princeton LN/CCD data collection system, with 514.5/568.2-nm excitation from a Spectra–Physics model 2060 argon–ion by Cobolt Lasers, Inc. Spectra in acetonitrile and deuterated acetonitrile were obtained at 77 K using a 135° backscattering geometry. The detector was cooled to -120 ^{o}C prior to the experiments. Spectral calibration was performed using the Raman spectrum of acetonitrile/toluene 50:50 (v:v). The collected data was processed using Spectragryph and a multipoint baseline correction was performed for all spectra.

Iron K-edge X-ray absorption spectra were collected on samples at SSRL beam line 9-3 using a 100 element solid state Ge detector (Canberra) with a SPEAR storage ring current of ~500 mA at a power of 3.0 GeV. The incoming X-rays were unfocused using a Si(220) double crystal monochromator, which was detuned by 30% of the maximal flux to attenuate harmonic X-rays. Seven scans were collected from 6882 eV to 8000 eV at a temperature (10 K) that was controlled by an Oxford Instruments CF1208 continuous flow liquid helium cryostat. An iron foil was placed in the beam pathway prior to I_0 and scanned concomitantly for an energy calibration, with the first inflection point of the edge assigned to 7112.0 eV. A 3, 6 or 9 μm Mn filter and a Soller slit were used to increase the signal-to-noise ratio of the spectra. Photoreduction was monitored by scanning the same spot on the sample twice and comparing the first derivative peaks associated with the edge energy during collection.

The detector channels from the scans were examined, calibrated, averaged, and processed for EXAFS analysis using EXAFSPAK to extract $\chi(k)$. Theoretical phase and amplitude parameters for a given absorber-scatterer pair were calculated using FEFF 8.40[3] and utilized by the "opt" program of the EXAFSPAK package during curve fitting.[4] Parameters for each species were calculated using a DFT model. In all analyses, the coordination number of a given shell was a fixed parameter and was varied iteratively in integer steps, while the bond lengths (R) and mean-square deviation (σ^2) were allowed to were allowed to freely float. Pre-edge analysis was performed on data normalized in the "process" program of the EXAFSPAK package, and pre-edge features were fit, as described elsewhere,[5] between 7108 eV to 7118 eV using the Fityk program with pseudo-Voigt functions composed of 50:50 Gaussian/Lorentzian functions.[5]

Computational Details. Computational studies were performed using ADF/AMS, Orca and QUILD,[6–9] as reported earlier. Briefly, geometry optimization and frequency calculations were performed using the unrestricted density functional r2scan-3c[10] with the corresponding def2-mTZVPP basis set. Free energy (ΔG) corrections, thermal and entropic corrections were made from frequency calculations at 298 K. Mössbauer and X-ray Absorption Spectroscopy calculations were performed using ADF/AMS, with the S12g density functional[11] and a large (all-electron) TZ2P basis set. The solvation energy was considered using acetonitrile as a solvent with the COSMO solvation model as implemented in ADF/AMS and Orca. All DFT calculations were performed using the unrestricted Kohn-Sham scheme. All computational data have been uploaded onto the IOCHEM-BD platform (www.iochem-bd.org) to facilitate data exchange and dissemination, according to the FAIR principles[12] of OpenData sharing.

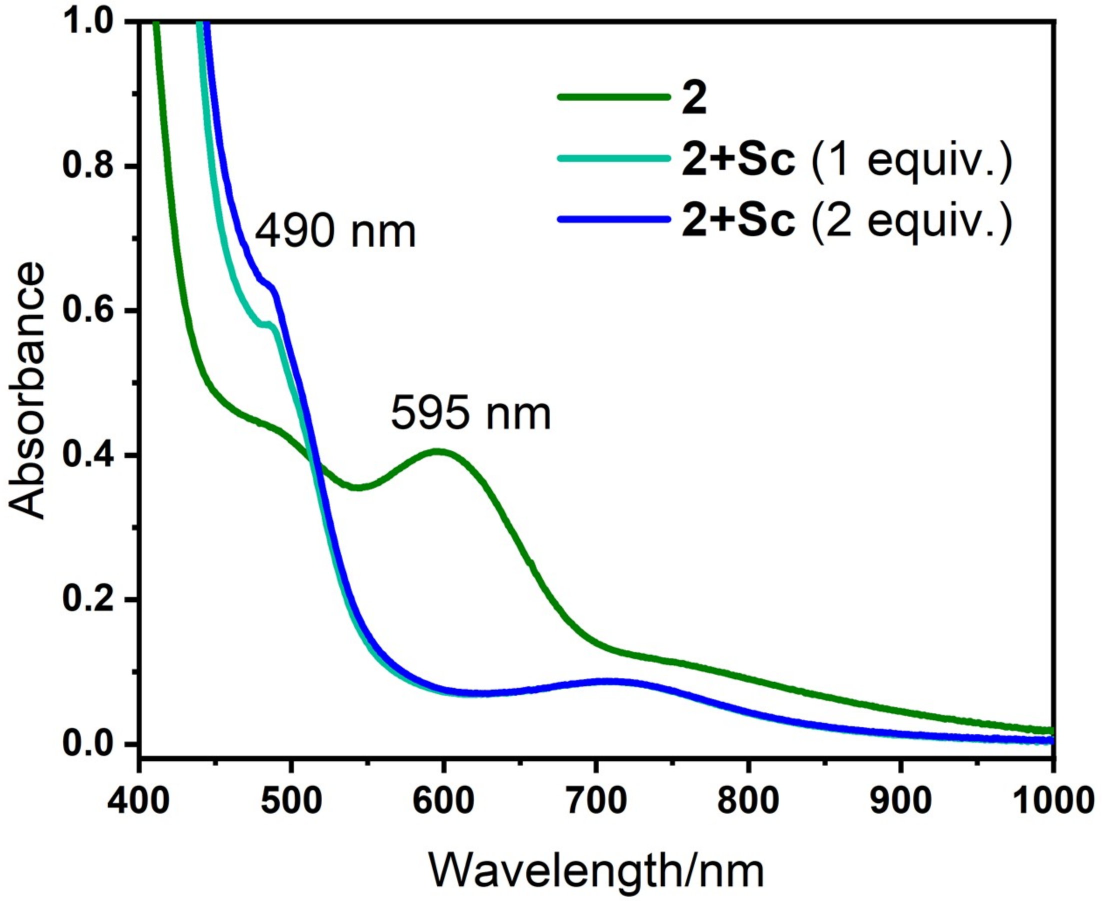

Figure S1. UV-vis spectral changes of **2** upon the addition of 1 equiv. and 2 equiv. of Sc^{3+} in MeCN at -40 ^{o}C, immediately after addition of Sc^{3+}

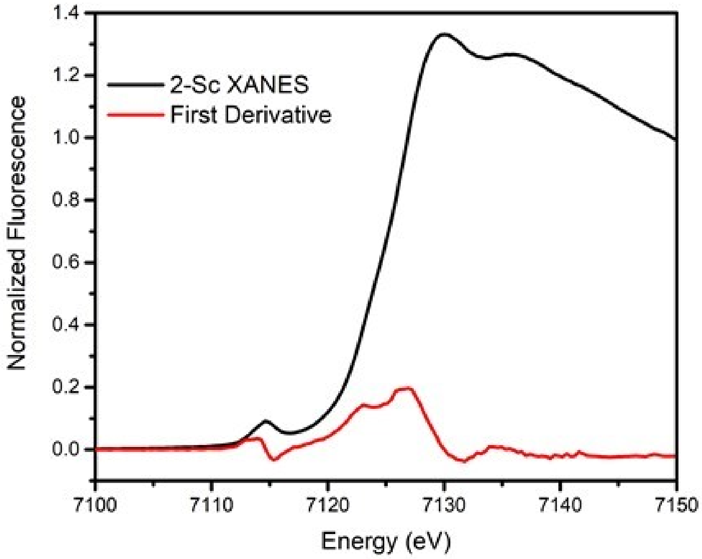

Figure S2. XANES spectra of **2+Sc**

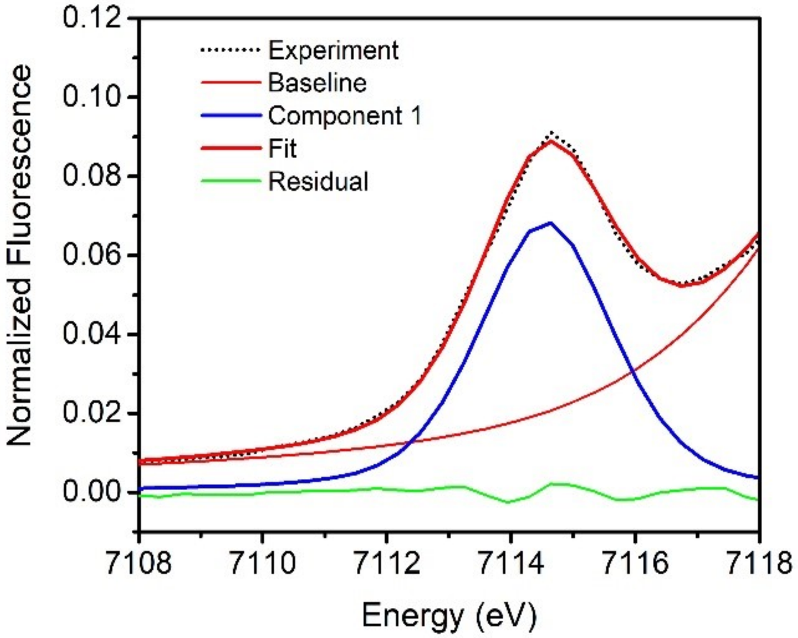

Figure S3. pre-edge spectrum of **2+Sc**, which is best fit with one feature in contrast to **2+H$^+$**.

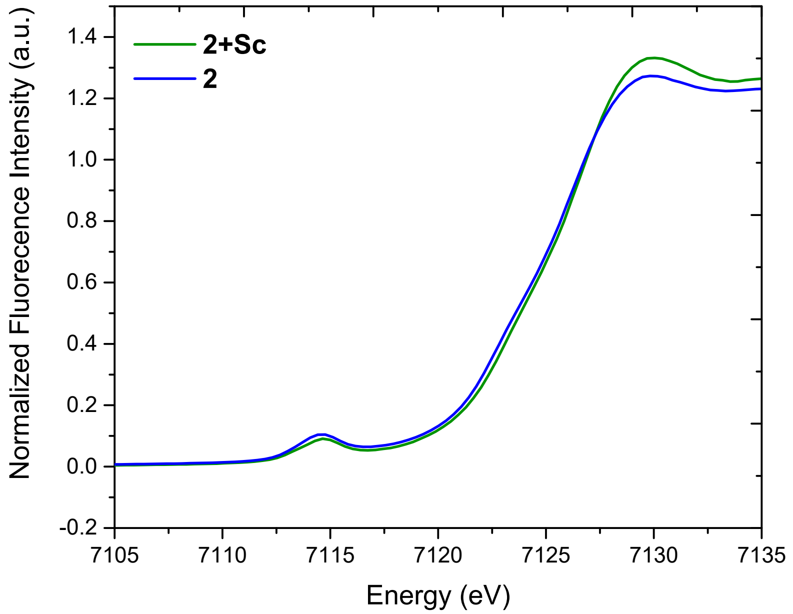

Figure S4. Comparison between **2** and **2+Sc** XANES spectra, notice the slight decrease in intensity in the pre-edge feature, and the small blue shift in the rising-edge feature upon the addition of 1 equiv Sc^{3+} to **2**.

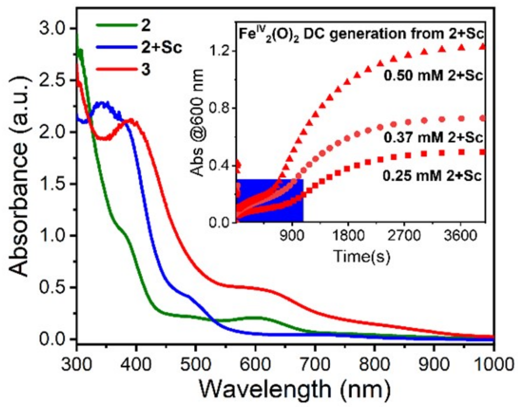

Figure S5. UV-Vis spectral changes at -40 °C upon treating 0.25-mM **2** in MeCN (green line) with 1 equiv Sc^{3+} to generate **2+Sc** (blue line), and with 2 equiv Sc^{3+} to generate **3** (red line). Inset shows time traces for the absorption band of diamond core (DC) complex **3** after addition of 2 equiv Sc^{3+} in three concentrations 0.25 mM, 0.37 mM, and 0.5 mM (red-dotted lines).

Although varying the concentration of the 2 equiv Sc^{3+} that is added does not change the kinetics of formation of **3**, the initial induction periods and the amount of **3** that is generated vary with the concentration of the 2 equiv Sc^{3+} (Figure S5, inset). Clearly there is an effect of varying the concentration of the 2 equiv Sc^{3+} but what it means remains unclear at the present time.

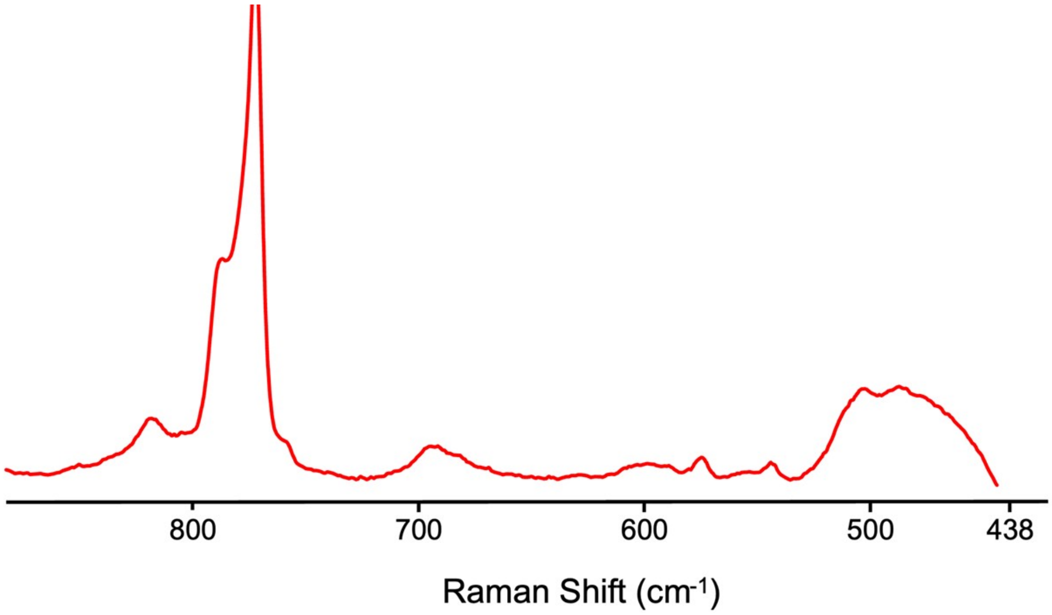

Figure S6. Resonance Raman spectrum of **2+Sc** including the region with vibrations associated with the Fe(μ-O)(μ-O_2)Fe five-membered ring.

Table S1. Mössbauer simulation parameters for various species shown in Figure 4.

		δ ($mm \bullet s^{-1}$)	ΔE_Q ($mm \bullet s^{-1}$)	Γ ($mm \bullet s^{-1}$)	%
Sim I, Fig 2A	Site A	0.48	1.54	0.40	46
	Site B	0.46	0.88	0.36	47
	Fe^{2+}	1.31	2.55	-0.40	4
Sim II, Fig 2B	Site 1	0.48	1.60	0.36	37
	Site 2	0.46	0.81	0.35	37
	Site 3	0.50	1.14	0.31	18
	Fe^{2+}	1.28	2.58	-0.40	4

Table S2. EXAFS fitting parameters for **2-Sc**, best fit in bold

2+Sc	Fe-N/O			Fe-O/N			Fe···C/N			Fe···Fe			Fe···Sc				GOF	
Fit	N	R(Å)	$\sigma^2(10^{-3})$	N	R(Å)	$\sigma^2(10^{-3})$	N	R(Å)	$\sigma^2(10^{-3})$	N	R(Å)	$\sigma^2(10^{-3})$	N	R(Å)	$\sigma^2(10^{-3})$	E_0	F	F'
**1**	4	2.10	5.16													-0.34	723	637
**2**	4	2.09	4.40	1	1.84	3.67										-3.42	591	635
**3**	4	2.09	4.41	1	1.84	3.75				1	3.16	8.48				-2.88	565	632
**4**	4	2.09	4.40	1	1.84	3.88				1	3.16	8.48	1	4.09	4.00	-2.43	537	629
**5**	5	2.09	4.39	1	1.84	3.65	3	2.97	4.34	1	3.16	5.08	1	4.09	4.16	-3.06	486	627
**6**	4	2.09	4.36	1	1.84	3.72	3	2.97	5.27	1	3.15	5.22				-2.89	504	627
							3	4.05	2.31									
**7**	**5**	**2.09**	**4.34**	**1**	**1.84**	**3.68**	**3**	**2.97**	**4.33**	**1**	**3.16**	**5.22**	**1**	**4.08**	**4.19**	**-3.49**	**451**	**627**
							**4**	**4.43**	**3.02**									

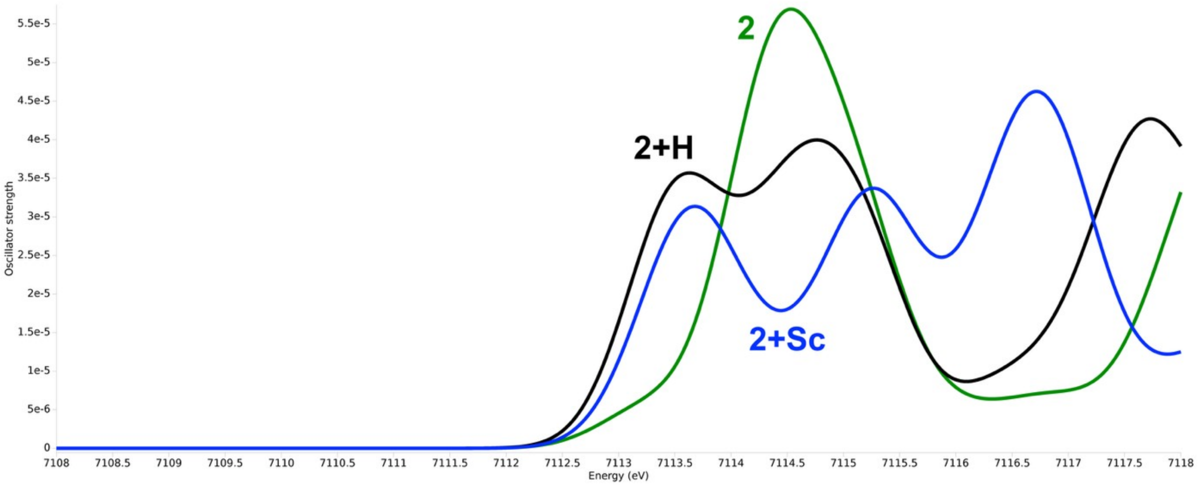

Figure S7. Computed XANES spectra (S12g/TZ2P, COSMO, ZORA) on r2scan-3c(COSMO) optimized geometries

Table S3. Computed Fe-O and O-O frequencies (cm^{-1})[a], geometric parameters (Å)[a], and Mössbauer parameters ($mm \cdot s^{-1}$)[b]

	**2**	**2+H**	**2+Sc**
ν(O-O)	913.1	934.6	882.0
ν_{asym}(Fe-O_2-Fe)	496.5	498.2	544.4
ν_{sym}(Fe-O_2-Fe)	439.2	437.7	454.8
ν_{asym}(Fe-O-Fe)	678.3	635.0	713.4
ν_{sym}(Fe-O-Fe)	513.7	509.9	501.2
R(O^{per}-O^{per})	1.410	1.394	1.465
R(Fe1-O^{oxo})	1.794	1.839	1.784
R(Fe2-O^{oxo})	1.837	1.827	1.820
R(Fe1-Fe2)	3.089	3.053	3.183
R(Fe1-O^{per1})	1.948	1.953	2.129
R(O^{per1}-Sc)	-	-	2.078
R(Fe1-Sc)	-	-	4.102
R(Fe2-O^{per2})	1.902	1.890	1.997
R(O^{per2}-Sc)	-	-	2.041
R(Fe2-Sc)	-	-	4.009
δ (Fe1)	0.475	0.446	0.412
δ (Fe2)	0.457	0.421	0.445
ΔE_Q (Fe1)	0.791	-0.807	-0.987
ΔE_Q (Fe2)	0.856	-0.701	-1.046

a) r2scan-3c/COSMO; b) S12g/TZ2P(ZORA,COSMO) at r2scan-3c/COSMO geometries

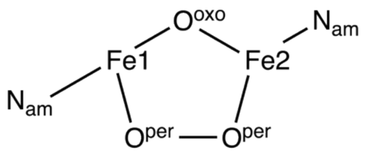

Cartesian coordinates (Å) for **2**

H	-5.75949909	1.59348156	0.04086794
H	-5.19692079	-2.78649756	2.17768620
O	-0.00150530	-0.95472127	1.56971236
Fe	0.00000000	0.00000000	0.00000000
H	-3.26375626	1.53368653	0.07437784
C	0.15704165	3.14571075	-4.78778484
C	-0.08940394	4.37820547	-4.19137989
C	0.62717232	0.12390430	-5.51030676
C	0.18372027	2.04341131	-3.94026053
C	-0.29996934	4.49692431	-2.80644939
N	0.40006292	0.69761420	-4.18862991
C	5.01899222	-1.90102944	-1.36276271
C	-0.02887504	2.14841849	-2.55153901
C	-0.27346635	3.39020911	-1.96557721
C	0.31021801	0.05444177	-2.99290944
C	0.45339545	-1.42868890	-2.84253412
N	0.05247305	0.88164871	-1.99385738
C	1.88180236	-2.28996200	-1.03455173
N	4.04804788	-0.93981398	-0.85323356
C	2.70208891	-1.07755788	-0.71700889
C	5.59162184	0.98160424	-0.26096159
N	0.52209696	-1.85486072	-1.42139112
C	4.37301531	0.32024896	-0.37412309
N	2.14058618	0.00000000	-0.19233120
C	5.55307625	2.25842459	0.28943119
C	3.15834863	0.90376324	0.03649480
C	-0.51862852	-2.85569703	-1.10554599
C	4.34551271	2.84844318	0.70607691
C	3.13054349	2.18544864	0.58538364
C	-1.82710110	-2.13906699	-0.98433055
N	-1.88167311	-0.86806174	-0.61317376
N	-3.06226917	-2.67107334	-1.18230619
C	-3.41423955	-4.03568715	-1.55873941
C	-3.22156579	-0.53567947	-0.55435772
C	-3.84651218	0.66081533	-0.20611084
C	-3.98061341	-1.66830388	-0.90837771
C	-5.23549271	0.68076490	-0.22720262
C	-5.37114915	-1.65492411	-0.93280026
C	-5.98359852	-0.45585799	-0.58429548
H	0.31893231	3.05470389	-5.85747816
H	-0.12024582	5.26938769	-4.81120555
H	-0.48899259	5.47985924	-2.38482095
H	-0.43406946	3.47391606	-0.89535687
H	0.80372831	-0.94832299	-5.42646634
H	-0.25039058	0.29260847	-6.14243634
H	1.50085954	0.59448434	-5.97128170
H	-0.40590450	-1.90965077	-3.33071369
H	1.34416589	-1.77229283	-3.38571868
H	2.35100188	-2.91478376	-1.80568046
H	1.80473613	-2.89488850	-0.12166312
H	4.52262141	-2.84123544	-1.60139898
H	5.49657008	-1.50418380	-2.26410146
H	5.78175746	-2.08602457	-0.60019348
H	6.52596480	0.52725825	-0.57614270
H	6.47980217	2.81333187	0.40181296
H	4.36730251	3.84726673	1.13242534
H	2.19287211	2.62927567	0.90670119
H	-0.28060607	-3.31069123	-0.13552402
H	-0.56214056	-3.66119832	-1.85191622
H	-2.51277676	-4.59599085	-1.80577550
H	-3.92687102	-4.52982035	-0.72733592
H	-4.07403904	-4.01481348	-2.43094584
H	-5.95230624	-2.53146480	-1.20226487
Fe	0.00000000	0.00000000	3.08900214
H	-7.06792169	-0.39643385	-0.58508015
O	-0.29255152	1.69211756	0.81772241
O	-0.11161510	1.73778500	2.21557601
H	-2.82926417	-2.00232332	2.15340057
C	-0.00928887	-4.54765864	6.53133848
C	-0.12137626	-5.50958656	5.53193543
C	0.25596654	-1.92818845	8.26517068
C	0.04316787	-3.22297513	6.11113903
C	-0.17947093	-5.15895223	4.17135474
N	0.14858119	-2.03264160	6.81408956
C	4.44162966	2.55808365	5.15496262
C	-0.01128234	-2.86054821	4.75061261

C	-0.12575953	-3.83289484	3.75797505
C	0.15474194	-1.02851760	5.89470024
C	0.27294796	0.42421551	6.24823678
N	0.06544166	-1.48341358	4.65779257
C	1.30094162	2.18621109	4.87780757
N	3.72915091	1.48814339	4.46608924
C	2.38849804	1.33178723	4.30329059
C	5.67733930	0.07820982	3.66010929
N	0.11470114	1.31893779	5.06997801
C	4.33978050	0.43293955	3.80342351
N	2.09343587	0.26603756	3.57480057
C	5.93401511	-1.09569058	2.95891461
C	3.29363460	-0.33747879	3.25790590
C	-1.15695033	2.07519735	5.11286653
C	4.89567259	-1.88358180	2.43018754
C	3.56282952	-1.51898375	2.56912831
C	-2.26030793	1.16537289	4.66950181
N	-2.04229914	0.21708999	3.77097128
N	-3.55735366	1.19561184	5.07925965
C	-4.19498865	2.11007819	6.01989025
C	-3.24702738	-0.42870010	3.57978381
C	-3.57832219	-1.51781864	2.77372527
C	-4.21995081	0.19036954	4.39017543
C	-4.89881540	-1.94633582	2.79778999
C	-5.54644950	-0.22942314	4.41209867
C	-5.86604931	-1.31089695	3.59771933
H	0.03362010	-4.82311285	7.58068404
H	-0.16556234	-6.55816046	5.81086143
H	-0.26853120	-5.94564895	3.42791336
H	-0.17094077	-3.55405082	2.70904401
H	0.33744008	-0.88065264	8.55573619
H	-0.63190409	-2.36242850	8.73502026
H	1.14641865	-2.46372883	8.60875904
H	-0.47106225	0.67730266	7.01620687
H	1.25582351	0.59112386	6.71084026
H	1.61517696	2.67064024	5.81284453
H	1.04198679	2.97114901	4.15712343
H	3.72776024	3.24962573	5.60220156
H	5.07573719	2.13502638	5.93991467
H	5.06466278	3.10368041	4.43917793
H	6.48109861	0.67856488	4.07514951
H	6.96289491	-1.41570185	2.82328736
H	5.14572140	-2.79915013	1.90186550
H	2.75575575	-2.12405745	2.16632795
H	-1.08618324	2.90428718	4.39833750
H	-1.35044092	2.50103045	6.10630458
H	-3.43819365	2.69802864	6.53871797
H	-4.87110610	2.78536514	5.48586276
H	-4.76423830	1.53355214	6.75457105
H	-6.29538152	0.25521839	5.03082684
H	-6.88873545	-1.67570408	3.58105213

Cartesian coordinates (Å) for **2+H**

H	-6.88348866	-0.85577477	0.70181010
H	-5.24793972	-2.61929302	4.14836609
O	-0.39737550	-0.93322978	1.51946975
Fe	0.00000000	0.00000000	0.00000000
H	-4.54947599	-0.68850505	1.55214622
C	-4.68458070	1.10861153	-2.80044726
C	-5.17427980	2.04783525	-1.89948844
C	-3.05681900	-1.02651004	-4.42200425
C	-3.43996013	0.56414143	-2.50558537
C	-4.44841367	2.42269339	-0.75544843
N	-2.68393087	-0.40651018	-3.15336316
C	4.57066883	-1.79203727	-2.43886865
C	-2.70578089	0.93360962	-1.36475286
C	-3.20562523	1.87318685	-0.46611696
C	-1.56561664	-0.59783791	-2.41845285
C	-0.44511548	-1.54024344	-2.71243485
N	-1.53406678	0.19043792	-1.34589347
C	1.60151634	-2.28225501	-1.62617583
N	3.68816987	-0.86571152	-1.73722947
C	2.39360553	-1.03867877	-1.39611665
C	5.31796676	1.00837795	-1.25039107
N	0.16202734	-1.99615200	-1.43483133
C	4.09015373	0.35856410	-1.22051504
N	1.91271885	0.00000000	-0.71547104
C	5.38197021	2.23454915	-0.59736292
C	2.96203822	0.90040221	-0.58058139
C	-0.50136186	-3.21647622	-0.88230654
C	4.26134391	2.78401897	0.04891996
C	3.03405322	2.13286772	0.06491268
C	-1.96345439	-3.01177567	-0.69417195
N	-2.47318729	-2.22365460	0.26082497
N	-2.96212110	-3.47321168	-1.47115867
C	-2.89532250	-4.44051180	-2.56880253
C	-3.83669531	-2.10305109	0.07444614
C	-4.80224981	-1.33875170	0.72223512
C	-4.15606292	-2.91793739	-1.02173578
C	-6.09668233	-1.44034154	0.23522289
C	-5.45618890	-3.03149151	-1.50399076
C	-6.41875645	-2.27375152	-0.85218742
H	-5.24779483	0.81669925	-3.68106288
H	-6.14344889	2.50119326	-2.08392166
H	-4.87382878	3.15863310	-0.07980722
H	-2.64314585	2.15443865	0.41849181
H	-2.32947470	-1.79330992	-4.68764611
H	-4.04644323	-1.48242916	-4.32437675
H	-3.08046489	-0.26504787	-5.20719654
H	-0.76053939	-2.38286107	-3.34188777
H	0.31635695	-0.98198494	-3.27264705
H	1.79968625	-2.71656339	-2.61571033
H	1.91968762	-3.02687689	-0.88460564
H	4.01427896	-2.67744685	-2.74520124
H	4.98404451	-1.30063356	-3.32430036
H	5.38611230	-2.09224138	-1.77327341
H	6.18406464	0.58030789	-1.74499951
H	6.32123519	2.77932244	-0.58572157
H	4.35834545	3.74353886	0.54842176
H	2.16577731	2.54946143	0.56478153
H	-0.05417555	-3.41666908	0.09765812
H	-0.32247114	-4.08692931	-1.52632354
H	-1.85937410	-4.68042776	-2.80052151
H	-3.42387781	-5.35015548	-2.27049454
H	-3.37388596	-4.00983788	-3.45153461
H	-5.70386380	-3.66405258	-2.34964992
O	-0.08790252	1.70307717	0.81485558
H	-7.44724531	-2.31807307	-1.19612193
O	-0.24407442	1.73946256	2.19980886
Fe	0.00000000	0.00000000	3.05292387
H	-3.01457473	-1.94152523	3.27637826
C	0.82897748	-4.53477870	6.36408493
C	0.37254873	-5.49000040	5.46120252
C	1.70310370	-1.92628167	7.90698423
C	0.80126066	-3.21334559	5.93189920
C	-0.09199292	-5.13622975	4.18187342
N	1.17453530	-2.02941265	6.55082029
C	5.07204249	2.08560402	3.79315693
C	0.33684057	-2.84836293	4.65351538

C	-0.11801924	-3.81338179	3.75640215
C	0.92998586	-1.02458047	5.66983761
C	1.20431353	0.42191814	5.96013432
N	0.43447402	-1.47469490	4.52849221
C	1.94066428	2.08576940	4.29847576
N	4.09130762	1.11750151	3.31460138
C	2.74498578	1.12989735	3.47821309
C	5.60944720	-0.53223615	2.13280872
N	0.79182354	1.32404688	4.84670879
C	4.39642177	-0.01374858	2.57185894
N	2.15599775	0.10603910	2.87480147
C	5.55306281	-1.73918735	1.44441921
C	3.16719054	-0.65394928	2.31971169
C	-0.33874176	2.19946361	5.23142707
C	4.33447259	-2.40381744	1.21697897
C	3.12530858	-1.87504633	1.64995355
C	-1.58103310	1.36843830	5.19705331
N	-1.70357295	0.37079789	4.32994333
N	-2.66426349	1.48188112	6.00654592
C	-2.91529727	2.45913922	7.06140660
C	-2.91609754	-0.23440751	4.60150657
C	-3.51155743	-1.36875358	4.05351095
C	-3.54343047	0.47094605	5.64761100
C	-4.75238516	-1.74219716	4.55420323
C	-4.78774160	0.10613467	6.15092010
C	-5.38235387	-1.01340787	5.57847686
H	1.18584341	-4.81135098	7.35135471
H	0.37432416	-6.53586964	5.75310947
H	-0.43873707	-5.91771818	3.51226380
H	-0.47551728	-3.53444792	2.76893861
H	2.05624362	-0.91132497	8.09061042
H	0.92292771	-2.17531927	8.63330363
H	2.54139351	-2.61919085	8.02242334
H	0.68784661	0.70813616	6.88683725
H	2.27771836	0.54527425	6.15965504
H	2.54256307	2.54824152	5.09208189
H	1.54963427	2.88084736	3.65278489
H	4.56235117	2.95115861	4.21634399
H	5.71212777	1.62747196	4.55359308
H	5.68675035	2.41712023	2.95089847
H	6.55295875	-0.02930289	2.32052278
H	6.47478663	-2.18615760	1.08394206
H	4.34245110	-3.35417256	0.69029294
H	2.18081362	-2.38787817	1.48972422
H	-0.42567116	3.00157472	4.48866847
H	-0.18302403	2.66569171	6.21277103
H	-2.06440382	3.13316772	7.15284739
H	-3.80682704	3.04339829	6.81462047
H	-3.07151706	1.94046641	8.01190672
H	-5.26920337	0.65734785	6.95267021
H	-6.35324339	-1.33886980	5.93958046
H	-1.86036255	-1.70584283	0.92586573
C	1.75293257	2.23487043	-3.50077035
N	0.87448301	1.48862769	-3.59563109
C	2.84628282	3.17469247	-3.39347648
H	3.01104327	3.65941192	-4.35987355
H	3.75831939	2.65110975	-3.09143287
H	2.60786951	3.93368945	-2.64331781

Cartesian coordinates (Å) for **2+Sc**

H	4.26581216	-3.83028288	1.41102882
H	4.89484602	2.90159195	1.58007702
Fe	0.00000000	0.00000000	0.00000000
Fe	0.00000000	0.00000000	3.18303896
H	2.14564386	-2.53064550	1.25820233
C	1.74260019	-2.87113950	-4.65486970
C	1.91619097	-4.11358308	-4.05704829
C	1.26404338	0.14196010	-5.36997201
C	1.20310505	-1.86949257	-3.85427339
C	1.57500908	-4.33291634	-2.71122973
N	0.97316974	-0.52713871	-4.10539925
C	-3.34713285	4.18947261	-0.90593462
C	0.81787508	-2.08466439	-2.51635890
C	1.02853518	-3.32797480	-1.92475692
C	0.48181060	0.00845428	-2.96130215
C	0.18972518	1.45387947	-2.75184165
N	0.33146806	-0.89071825	-1.99525550
C	-0.49526082	2.87157744	-0.90555158
N	-3.02998604	2.77385144	-0.74065976
C	-1.81254911	2.18641196	-0.73684242
C	-5.35081258	1.81192221	-0.43223487
N	0.46455821	1.83011793	-1.33533145
C	-3.96547845	1.76954733	-0.53582350
N	-1.88433115	0.87016369	-0.55160138
C	-5.98798019	0.60261531	-0.18104532
C	-3.23168759	0.57369338	-0.41213863
C	1.88574535	2.21162510	-1.12343551
C	-5.26681993	-0.59673517	-0.04777291
C	-3.88333612	-0.63264402	-0.16718178
C	2.67331967	0.99544528	-0.74734775
N	2.11257225	0.00000000	-0.07506915
N	3.99079675	0.77349777	-0.97745853
C	4.96132895	1.64699836	-1.62744134
C	3.10264070	-0.93612989	0.15307676
C	3.06523025	-2.16099429	0.81693843
C	4.30078834	-0.45256881	-0.40445413
C	4.25405763	-2.87277045	0.89808061
C	5.49519008	-1.16105978	-0.32625327
C	5.44685297	-2.38234894	0.33605307
H	2.02992040	-2.69089594	-5.68599154
H	2.33898165	-4.92754289	-4.63823047
H	1.74915236	-5.31130456	-2.27293858
H	0.79302648	-3.49616578	-0.88374334
H	0.81024715	1.13242241	-5.38067204
H	2.34633051	0.22959995	-5.50859686
H	0.83920293	-0.44437557	-6.18918191
H	0.77300490	2.08473429	-3.43308008
H	-0.86957400	1.63942601	-2.96820295
H	-0.55571509	3.69887988	-1.62701538
H	-0.16515006	3.28223113	0.05620830
H	-2.42703134	4.77355582	-0.92474068
H	-3.89726460	4.34082240	-1.83947906
H	-3.96032503	4.52078346	-0.06297112
H	-5.90654719	2.73927561	-0.52946101
H	-7.06916677	0.58376567	-0.08335134
H	-5.80798380	-1.51579109	0.15362490
H	-3.32816121	-1.55956903	-0.07878470
H	1.93456486	2.92346653	-0.28944829
H	2.30097504	2.71495464	-2.00428505
H	4.45264407	2.49485087	-2.08547764
H	5.68066011	2.01475807	-0.88876642
H	5.49010147	1.08533387	-2.40259768
H	6.41935356	-0.78057853	-0.74993746
O	-0.19791072	-1.90471241	2.25208800
H	6.35504579	-2.97100374	0.42489984
O	-0.06819728	0.84263116	1.61200359
O	-0.40195080	-1.78275787	0.80607877
H	2.55308475	2.16642580	2.04211114
C	-0.41374156	4.75843889	6.18276048
C	-0.71023536	5.60308601	5.11799673
C	0.28106430	2.37151082	8.11055191
C	-0.17262968	3.42632568	5.86517845
C	-0.76424844	5.13239239	3.79390543
N	0.13044539	2.32841711	6.65903425
C	-3.79432645	-1.31684594	6.90304140
C	-0.21826706	2.94698933	4.54288596

C	-0.52085275	3.80010395	3.48410248
C	0.25203324	1.26077623	5.83243886
C	0.60878406	-0.12529774	6.27016322
N	0.06190701	1.59217843	4.56276919
C	-0.97975234	-1.84373821	5.50357492
N	-3.25922600	-0.69488023	5.69729239
C	-2.08826380	-0.94343860	5.06425368
C	-5.17212085	0.86452423	5.12230925
N	0.29504961	-1.14167065	5.22422350
C	-3.93819757	0.24962975	4.94068213
N	-1.96245321	-0.22900600	3.94906666
C	-5.52970837	1.82171446	4.17897851
C	-3.10035110	0.55048461	3.85072777
C	1.44175143	-2.05787178	5.00997366
C	-4.68548646	2.15129192	3.10342325
C	-3.46009571	1.52428407	2.92225538
C	2.45813789	-1.27223543	4.24354460
N	2.07318068	-0.24033306	3.49827901
N	3.80436125	-1.38680451	4.29519384
C	4.59645121	-2.41301053	4.96434357
C	3.22668962	0.39579493	3.07872176
C	3.40622758	1.58218503	2.37244611
C	4.33086722	-0.33438637	3.55597144
C	4.71350275	1.98079247	2.12697395
C	5.64224786	0.05056836	3.30179888
C	5.81084297	1.22214755	2.57173986
H	-0.37753426	5.12285456	7.20456762
H	-0.90762173	6.65238725	5.31531340
H	-1.00445766	5.82860624	2.99585358
H	-0.57095660	3.42578388	2.46669113
H	0.62169703	1.40364282	8.47754398
H	1.02227734	3.13131377	8.37454818
H	-0.67768484	2.62105622	8.57530571
H	1.68548132	-0.14810305	6.48695136
H	0.09681058	-0.37720294	7.20794847
H	-1.06968102	-2.13373196	6.55702409
H	-1.01449208	-2.76154561	4.90667948
H	-3.03617090	-1.94667848	7.36676324
H	-4.09541500	-0.53504051	7.60610879
H	-4.65973559	-1.93391783	6.64378547
H	-5.82158938	0.61959292	5.95675879
H	-6.48235592	2.33290621	4.27943265
H	-5.00114232	2.91228619	2.39633387
H	-2.79869073	1.77949122	2.10096606
H	1.10123758	-2.92023148	4.42890503
H	1.85447731	-2.43710607	5.95493133
H	3.93626781	-3.10390996	5.48878951
H	5.17411109	-2.96864348	4.21913005
H	5.27787437	-1.94328023	5.67960526
H	6.49103105	-0.52340529	3.66009085
H	6.81670999	1.56699567	2.35146970
Sc	-1.26642672	-3.50725351	1.47331204
O	-1.74919319	-5.87613406	-1.74014417
S	-4.03550446	-3.48071979	3.52389388
O	-3.31016632	-3.66276811	4.78930783
O	-3.10874701	-3.18070242	2.34297224
S	0.70874419	-5.21130773	2.56291971
O	-1.83270245	-3.57841630	-0.65987938
F	-4.77337039	-6.01264021	-0.99036331
O	-5.20678320	-2.59653165	3.50076997
O	-0.55597807	-4.73666415	3.24826961
F	-4.23840474	-4.50791298	-2.47485121
O	0.59330106	-4.70544668	1.13754667
C	-4.15762675	-4.84529874	-1.18539300
O	1.97641198	-4.96633553	3.24599480
F	1.51998685	-7.54892960	1.63168134
F	0.74415177	-7.61334530	3.66908269
F	-4.74149058	-3.90076830	-0.44722188
O	-2.29029147	-5.49374680	0.71105163
F	-3.70558916	-6.11141603	3.21108634
F	-0.62680333	-7.45916554	1.98658139
S	-2.32908902	-5.01619711	-0.71356855
F	-5.61172091	-5.51771195	4.08038513
C	0.56545753	-7.10231641	2.44993423
F	-5.27050180	-5.27263136	1.94501293
C	-4.69621091	-5.21705052	3.14885603

References:

(1) Seo, M. S.; Kim, N. H.; Cho, K.-B.; So, J. E.; Park, S. K.; Clémancey, M.; Garcia-Serres, R.; Latour, J.-M.; Shaik, S.; Nam, W. A Mononuclear Nonheme Iron(Iv)-Oxo Complex Which Is More Reactive than Cytochrome P450 Model Compound I. *Chem. Sci.* **2011**, *2* (6), 1039.

(2) Banerjee, S.; Draksharapu, A.; Crossland, P. M.; Fan, R.; Guo, Y.; Swart, M.; Que, L. Sc3+-Promoted O-O Bond Cleavage of a (μ-1,2-Peroxo)Diiron(III) Species Formed from an Iron(II) Precursor and O2 to Generate a Complex with an FeIV2(μ-O)2Core. *J. Am. Chem. Soc.* **2020**, *142*, 4285–4297.

(3) George, G. N. EXAFSPAK. Stanford Synchrotron Radiation Laboratory 1990.

(4) Ankudinov, A.; Ravel, B.; Rehr, J.; Conradson, S. Real-Space Multiple-Scattering Calculation and Interpretation of x-Ray-Absorption near-Edge Structure. *Phys. Rev. B* **1998**, *58*, 7565–7576.

(5) Westre, T. E.; Kennepohl, P.; DeWitt, J. G.; Hedman, B.; Hodgson, K. O.; Solomon, E. I. A Multiplet Analysis of Fe K-Edge 1s → 3d Pre-Edge Features of Iron Complexes. *J. Am. Chem. Soc.* **1997**, *119* (27), 6297–6314.

(6) E.J. Baerends, T. Ziegler, A.J. Atkins, J. Autschbach, O. Baseggio, D. Bashford, A. Bérces, F.M. Bickelhaupt, C. Bo, P.M. Boerrigter, C. Cappelli, L. Cavallo, C. Daul, D.P. Chong, D.V. Chulhai, L. Deng, R.M. Dickson, J.M. Dieterich, F. Egidi, D.E. Ellis, M. van Faassen, L. Fan, T.H. Fischer, A. Förster, C. Fonseca Guerra, M. Franchini, A. Ghysels, A. Giammona, S.J.A. van Gisbergen, A. Goez, A.W. Götz, J.A. Groeneveld, O.V. Gritsenko, M. Grüning, S. Gusarov, F.E. Harris, P. van den Hoek, Z. Hu, C.R. Jacob, H. Jacobsen, L. Jensen, L. Joubert, J.W. Kaminski, G. van Kessel, C. König, F. Kootstra, A. Kovalenko, M.V. Krykunov, P. Lafiosca, E. van Lenthe, D.A. McCormack, M. Medves, A. Michalak, M. Mitoraj, S.M. Morton, J. Neugebauer, V.P. Nicu, L. Noodleman, V.P. Osinga, S. Patchkovskii, M. Pavanello, C.A. Peeples, P.H.T. Philipsen, D. Post, C.C. Pye, H. Ramanantoanina, P. Ramos, W. Ravenek, M. Reimann, J.I. Rodríguez, P. Ros, R. Rüger, P.R.T. Schipper, D. Schlüns, H. van Schoot, G. Schreckenbach, J.S. Seldenthuis, M. Seth, J.G. Snijders, M. Solà, M. Stener, M. Swart, D. Swerhone, V. Tognetti, G. te Velde, P. Vernooijs, L. Versluis, L. Visscher, O. Visser, F. Wang, T.A. Wesolowski, E.M. van Wezenbeek, G. Wiesenekker, S.K. Wolff, T.K. Woo, A.L. Yakovlev, ADF 2025.1, SCM, Theoretical Chemistry, Vrije Universiteit, Amsterdam, The Netherlands, http://www.scm.com, 2025.

(7) E.J. Baerends, N.F. Aguirre, N.D. Austin, J. Autschbach, F.M. Bickelhaupt, R. Bulo, C. Cappelli, A.C.T. van Duin, F. Egidi, C. Fonseca Guerra, A. Förster, M. Franchini, F.T.P.M. Goumans, T. Heine, M. Hellström, C.R. Jacob, L. Jensen, M. Krykunov, E. van Lenthe, A. Michalak, M.M. Mitoraj, J. Neugebauer, V.P. Nicu, P. Philipsen, H. Ramanantoanina, R. Rüger, G. Schreckenbach, M. Stener, M. Swart, J.M. Thijssen, T. Trnka, L. Visscher, A. Yakovlev, and S.J.A. van Gisbergen, The Amsterdam Modeling Suite, J. Chem. Phys. 2025, 162, 162501.

(8) Swart, M.; Bickelhaupt, F. M. QUILD: QUantum-Regions Interconnected by Local Descriptions. J. Comput. Chem. 2008, 29, 724−734.

(9) F. Neese, Software Update: The ORCA Program System—Version 6.0, WIREs Comp. Mol. Sci. 2025, 15, e70019.

(10) S. Grimme, A. Hansen, S. Ehlert, J-M. Mewes, r2SCAN-3c: A "Swiss army knife" composite electronic-structure method, J. Chem. Phys. 2021, 154, 064103.

(11) Swart, M. A. New Family of Hybrid Density Functionals. Chem. Phys. Lett. 2013, 580 (0), 166−171.

(12) M. D. Wilkinson, M. Dumontier, I. J. Aalbersberg, G. Appleton, M. Axton, A. Baak, N. Blomberg, J.-W. Boiten, L. B. da Silva Santos, P. E. Bourne, J. Bouwman, A. J. Brookes, T. Clark, M. Crosas, I. Dillo, O. Dumon, S. Edmunds, C. T. Evelo, R. Finkers, A. Gonzalez- Beltran, A. J. G. Gray, P. Groth, C. Goble, J. S. Grethe, J. Heringa, P. A. C. 't Hoen, R. Hooft, T. Kuhn, R. Kok, J. Kok, S. J. Lusher, M. E. Martone, A. Mons, A. L. Packer, B. Persson, P. Rocca-Serra, M. Roos, R. van Schaik, S.-A. Sansone, E. Schultes, T. Sengstag, T. Slater, G. Strawn, M. A. Swertz, M. Thompson, J. van der Lei, E. van Mulligen, J. Velterop, A. Waagmeester, P. Wittenburg, K. Wolstencroft, J. Zhao and B. Mons, Scientific Data, 3, 160018 (2016)
